# Resistance exercise for optimizing cognitive function during aging: the role of individual characteristics

**DOI:** 10.1002/alz70860_102755

**Published:** 2025-12-23

**Authors:** Patricio Solis‐Urra, Beatriz Fernandez‐Gamez, Andrea Coca‐Pulido, Cristina Molina‐Hidalgo, Marcos Olvera‐Rojas, Esmée A Bakker, Dario Bellon, Alessandro Sclafani, Jose Mora‐Gonzalez, Javier Fernandez‐Ortega, Lucía Sánchez‐Aranda, Isabel Martin‐Fuentes, Angel Toval, Lu Wan, Manuel Gomez‐Rio, Teresa Liu‐Ambrose, Kirk I. Erickson, Francisco B Ortega, Irene Esteban‐Cornejo

**Affiliations:** ^1^ Faculty of Sport Sciences, Sport and Health University Research Institute (iMUDS), University of Granada., Granada, Granada, Spain; ^2^ AdventHealth Research Institute, Orlando, FL, USA; ^3^ Andrés Bello University, Valparaiso, Chile; ^4^ University of Pittsburgh, Pittsburgh, PA, USA; ^5^ Servicio de Medicina Nuclear, Hospital Universitario Virgen de las Nieves, 18014, Granada, España, GRANADA, Granada, Spain; ^6^ Centre for Aging SMART, Vancouver Coastal Health Research Institute, Vancouver, BC, Canada; ^7^ University of British Columbia, Vancouver, BC, Canada; ^8^ Djavad Mowafaghian Centre for Brain Health, Vancouver, BC, Canada; ^9^ Faculty of Sport and Health Sciences University of Jyväskylä., Jyväskylä, Finland; ^10^ CIBER de Fisiopatología de la Obesidad y Nutrición (CIBEROBN), Instituto de Salud Carlos III, Granada, Andalucia, Spain; ^11^ Instituto de Investigación Biosanitaria (ibs.GRANADA), Granada, Spain

## Abstract

Physical exercise has emerged as one of the leading non‐pharmaceutical approaches to delay cognitive decline. However, the limited understanding of the mechanisms by which exercise impacts cognition and the poor description of exercise programs in late adulthood hinder the widespread use of exercise as a therapeutic or preventive approach for Alzheimer's Disease (AD), with resistance exercise (RE) being one of the understudied types of exercise. Despite their potential significance, few studies with well‐characterized samples and comprehensive cognitive assessments have attempted to understand the role of key demographics and clinical variables on the effect of resistance exercise on cognition. The AGUEDA trial aims to uncover the effects of a 24‐week RE intervention on cognitive performance, depending on individual participant's characteristics.

The AGUEDA trial is a single‐site, two‐arm, single‐blinded, randomized controlled trial involving 90 cognitively normal older adults (71.75 ± 3.96 years, 57% female) from Spain. Participants were randomized to a RE group (*n* = 46) or control group (*n* = 44). The exercise group performed 180 minutes/week of supervised elastic band and body weight resistance exercises for 24 weeks, while controls maintained usual activities. High adherence was observed, and no serious adverse events occurred. During this talk, we will share promising findings on the impact of exercise on executive function and cognitive subdomains such as attentional/inhibitory control, episodic memory, processing speed, visuospatial memory and working memory, with a focus on optimizing the effect based on individual participant's characteristics. Demographics and key clinical characteristics such as sex, age, education level, number of comorbidities, apolipoprotein E carrier, Amyloid burden, baseline cognitive performance of each cognitive outcome and subjective cognitive decline are explored as potential moderators of the exercise's impact on cognition. Additional mechanisms related to physical parameters are also explored as potential mediators of cognitive changes.

This research demonstrates the selective potential of RE as a powerful tool in the new era of precision interventions targeting specific AD‐related cognitive decline. It also provides valuable insights into optimizing benefits based on patient characteristics, necessitating a thorough exploration of the optimal types and doses of exercise before advancing to comprehensive interventions.

